# Vegetation Heterogeneity Effects on Soil Macro-Arthropods in an Alpine Tundra of the Changbai Mountains, China

**DOI:** 10.3390/plants8100418

**Published:** 2019-10-16

**Authors:** Yan Tao, Zhongqiang Wang, Chen Ma, Hongshi He, Jiawei Xu, Yinghua Jin, Haixia Wang, Xiaoxue Zheng

**Affiliations:** 1 Key Laboratory of Geographical Processes and Ecological Security in Changbai Mountains, Ministry of Education, School of Geographical Sciences, Northeast Normal University, Changchun 130024, Jilin, China; taoy431@nenu.edu.cn (Y.T.); wangzq027@nenu.edu.cn (Z.W.); hehs100@nenu.edu.cn (H.H.); xujw634@nenu.edu.cn (J.X.); jinyh796@nenu.edu.cn (Y.J.); wanghx885@nenu.edu.cn (H.W.); zhengxx856@nenu.edu.cn (X.Z.); 2 School of Public Administration and Law, Northeast Agricultural University, Harbin 150030, Heilongjiang, China; 3 School of Natural Resources, University of Missouri, Columbia, MO 65211, USA

**Keywords:** soil macro-arthropods, vegetation heterogeneity, taxonomic composition, diversity, alpine tundra, Changbai Mountains

## Abstract

The harsh environmental conditions in alpine tundra exert a significant influence on soil macro-arthropod communities, yet few studies have been performed regarding the effects of vegetation heterogeneity on these communities. In order to better understand this question, a total of 96 soil macro-arthropod samples were collected from four habitats in the Changbai Mountains in China, namely, the *Vaccinium uliginosum* habitat, *Sanguisorba sitchensis* habitat, *Rhododendron aureum* habitat, and *Deyeuxia angustifolia* habitat. The results revealed that the taxonomic composition of the soil macro-arthropods varied among the habitats, and that dissimilarities existed in these communities. The abundance, richness and diversity in the *D. angustifolia habitat* were all at their maximum during the sampling period. The vegetation heterogeneity affected the different taxa of the soil macro-arthropods at various levels. In addition, the vegetation heterogeneity had direct effects not only on soil macro-arthropod communities, but also indirectly impacted the abundance, richness and diversity by altering the soil fertility and soil texture. Overall, our results provide experimental evidence that vegetation heterogeneity can promote the abundance, richness and diversity of soil macro-arthropods, yet the responses of soil macro-arthropods to vegetation heterogeneity differed among their taxa.

## 1. Introduction

The “habitat heterogeneity hypothesis” [1] states that the number of available ecological niches will increase as habitats gradually become more complex, which will have positive effects on the abilities of species to coexist [2]. This hypothesis indicates that vegetation heterogeneity will increase diversity, which will cause a range of cascading changes in ecological processes and functions, and is thus crucial for maintaining the stability of the ecosystem [3,4]. Therefore, it is necessary to provide some insight into elucidating the effects of vegetation heterogeneity on the ecosystem.

Soil arthropods play crucial roles in nutrient cycling and energy flows of belowground ecosystems, and participate in soil ecosystem services [5]. Soil arthropods can increase nutrient mineralization via physical fragmentation [6], excreting faeces [7], and microbial modification [8]. At the same time, soil arthropods are able to respond sensitively to environmental changes due to the relative decrease in their activities [9]. Consequently, soil arthropods, as indicator species, have become an important topic in the study of belowground systems. In general, the distribution patterns and diversity characteristics of soil arthropods are affected by various factors (such as plant communities, litter and soil properties), and the evidence of relationships between soil arthropods and these factors is substantial [10,11]. However, there is a relatively small number of studies regarding the effects of vegetation heterogeneity on soil macro-arthropods available at present.

Alpine tundra is a relatively harsh type of ecosystem, and has a low level of biodiversity as it is characterized by low temperatures and high elevation [12]. It features the presence of snow on the ground and high solar radiation for the greatest part of the year, thus the environment in alpine tundra is unique and fragile [13]. Its specific environmental conditions exert a significant influence on soil macro-arthropod communities. Nevertheless, studies regarding alpine tundra soil macro-arthropods are relatively rare and not highly generalized. The majority of the current studies mainly focus on their distribution and response to climate change, and most of these studies have been performed in Colorado and European alpine tundra [14,15]. In contrast, there is a lack of soil macro-arthropods data collection in the alpine tundra of East Asia, and in particular there is a lack of information regarding the effects of alpine tundra vegetation heterogeneity on soil macro-arthropods.

In the present study, in order to better understand the effects of the vegetation heterogeneity on soil macro-arthropod communities in the alpine tundra, we selected the tundra of the Changbai Mountains as the experimental site, as it is one of the two alpine tundra areas in China. The soil macro-arthropods were collected from four different habitats (i.e., the *Vaccinium uliginosum* habitat, *Sanguisorba sitchensis* habitat, *Rhododendron aureum* habitat and *Deyeuxia angustifolia* habitat) in the alpine tundra of the Changbai Mountains. In this study, we hypothesized the following: (H1) the distribution patterns of soil macro-arthropods differ in each plant community caused by vegetation heterogeneity; (H2) there are similarities in the structure of the soil macro-arthropod communities among the different sampling sites; and (H3) vegetation heterogeneity affects the different taxa of soil macro-arthropods at various levels.

## 2. Results

### 2.1. Taxonomic Composition of Soil Macro-Arthropod Communities

During the study period, a total of 2848 soil macro-arthropods belonging to 28 taxa (families/suborders) and 11 orders were collected from the four habitats. The dominant taxa were found to be Carabidae (15.17%), Lithobiidae (11.24%), and Elateridae (10.11%). Seventeen taxa were considered to be common and accounted for 58.99% of the total number. In addition, the other eight taxa were rarely collected and accounted for 4.49% of the total number of soil macro-arthropods.

**Figure 1:**
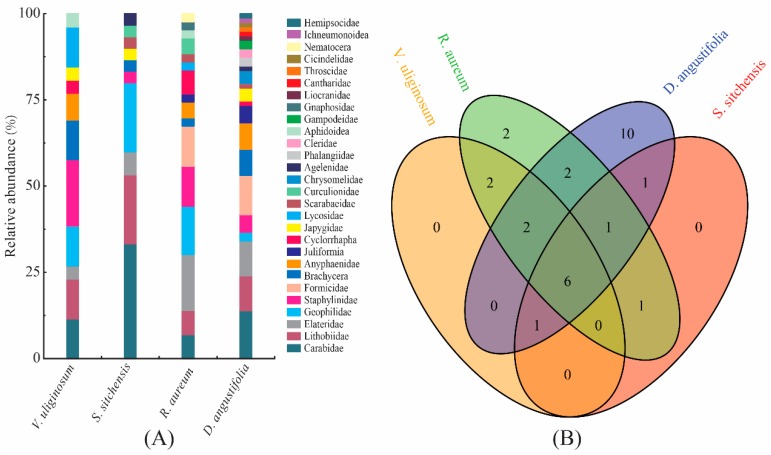
Soil macro-arthropods community composition in the habitats. (**A**) Relative abundance of the soil macro-arthropods community; (**B**) Venn diagram of the number of shared and unique arthropod taxa in the different habitats. Shared and unique numbers in the circles indicate either unique number of taxa or number of shared taxa in the overlap regions.

The taxonomic composition of soil macro-arthropods from different habitats is displayed in Figure 1A. In the *V. uliginosum* habitat, Carabidae, Lithobiidae, Geophilidae, Staphylinidae, Brachycera and Lycosidae were the dominant taxa, together accounting for 76.93% of the total number of soil macro-arthropods. Carabidae (33.33%), Lithobiidae (20.00%) and Geophilidae (20.00%) were collected frequently and accounted for 73.33% of the total number of soil macro-arthropods in the *S. sitchensis* habitat. The dominant taxa were found to be Elateridae (16.28%), Geophilidae (13.95%), Staphylinidae (11.63%) and Formicidae (11.63%) in the *R. aureum* habitat. Carabidae (13.92%), Lithobiidae (10.13%), Elateridae (10.13%) and Formicidae (11.39%) were considered to be the dominant taxa in the *D. angustifolia* habitat.

Venn diagrams with unique and shared taxa are detailed in Figure 1B. The four habitats had six taxa (Carabidae, Lithobiidae, Elateridae, Geophilidae, Staphylinidae and Brachycera) in common, which contributed to between 26.09% and 60.00% of the full set of taxa in each habitat. Gnaphosidae and Nematocera were found only in the *R. aureum* habitat, while Chrysomelidae, Phalangiidae, Cleridae, Gampodeidae, Liocranidae, Cantharidae, Throscidae, Cicindelidae, Ichneumonoidea and Hemipsocidae were observed only in the *D. angustifolia* habitat. Unique taxa were not found in the *V. uliginosum* or *S. sitchensis* habitats.

### 2.2. Diversity Characteristics of Soil Macro-Arthropods

**Figure 2:**
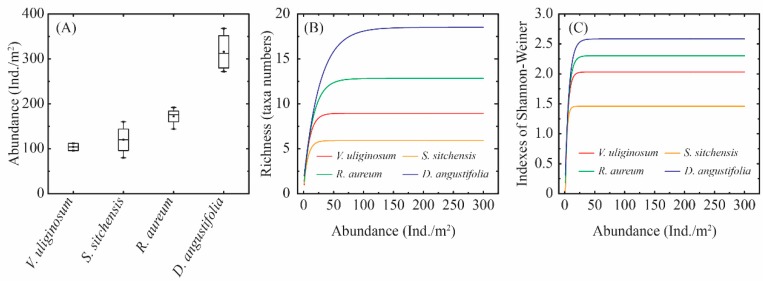
Diversity characteristics of soil macro-arthropods in different habitats. (**A**–**C**) Box-Whisker plots illustrating the medians (line in the box), 25th and 75th percentiles (box), 10th and 90th percentiles (outer lines), and mean values (dots) of abundance (**A**) of soil macro-arthropods in the different habitats. (**B**,**C**) Rarefaction curves of soil macro-arthropods richness and Shannon-Wiener index assemblage in the various habitats.

During the study period, the diversity characteristics of the soil macro-arthropod communities were found to be different for each of the four habitats. The abundances of soil macro-arthropods are summarized in Figure 2A and Supplementary Materials Table S1. In the *V. uliginosum* habitat, 104 individuals/m^2^ of arthropods were collected; 120 individuals/m^2^ in the *S. sitchensis* habitat; 172 individuals/m^2^ in the *R. aureum* habitat; and 316 individuals/m^2^ in the *D. angustifolia* habitat. Among these habitats, the *D. angustifolia* habitat displayed the maximum abundance, and it was significantly higher than in the other habitats (*p* < 0.05). The minimum was found in the *V. uliginosum* habitat, while there were no significant differences observed among the *V. uliginosum*, *S. sitchensis* and *R. aureum* habitats.

The richness of soil macro-arthropods is detailed in Figure 2B. The curves were plateaus for all of the habitats, which demonstrates that the majority of soil macro-arthropods taxa were detected. The rarefaction curves indicate that the richness was different among the four habitats, the scale of which was as follows: *S. sitchensis* > *D. angustifolia* > *R. aureum* > *V. uliginosum.* Similar to richness, the rarefaction curves of the Shannon-Wiener indexes were also plateaus for all of the habitats, and it was shown that the diversity of soil macro-arthropods was accurately evaluated (Figure 2C). The diversity was different among the four habitats, and the trends of diversity levels were the same as the richness.

**Figure 3:**
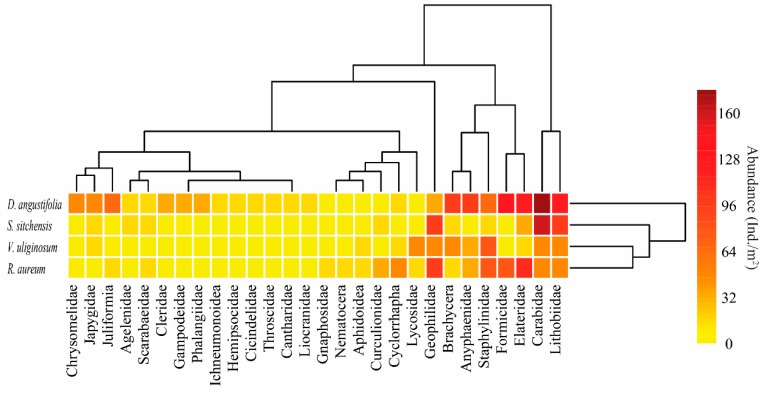
Abundance heatmap of log (x+1)-normalized soil macro-arthropods in the various habitats; dendrogram of sample sites based on similarity along right axis; dendrogram of soil macro-arthropods taxa based on similarity along upper axis. The colors represent the abundance of soil macro-arthropods.

A heat map showed that the four sampling sites could be divided into two clusters, indicating that major similarities exist among the levels in the *V. uliginosum*, *S. sitchensis* and *R. aureum* habitats. However, it was also revealed that a major dissimilarity existed between these habitats and the *D. angustifolia* habitat (Figure 3). At the same time, the soil macro-arthropods taxa could be divided into four clusters: Carabidae and Lithobiidae made up one cluster; Elateridae, Formicidae, Staphylinidae, Anyphaenidae, Brachycera were a second cluster; Geophilidae composed the third; and the fourth cluster consisted of all other taxa. A greater number of Carabidae and Lithobiidae were found in the *D. angustifolia* and *S. sitchensis* habitats, and the distribution patterns of Geophilidae were mainly found in the *S. sitchensis* and *R. aureum* habitats. Elateridae, Formicidae, Staphylinidae, Anyphaenidae and Brachycera were abundant in the *V. uliginosum* habitat. Additionally, most of the soil macro-arthropods taxa displayed evident evenness among most of the sampling sites.

### 2.3. Correlation of Soil Macro-Arthropod Communities to Vegetation Heterogeneity

**Figure 4:**
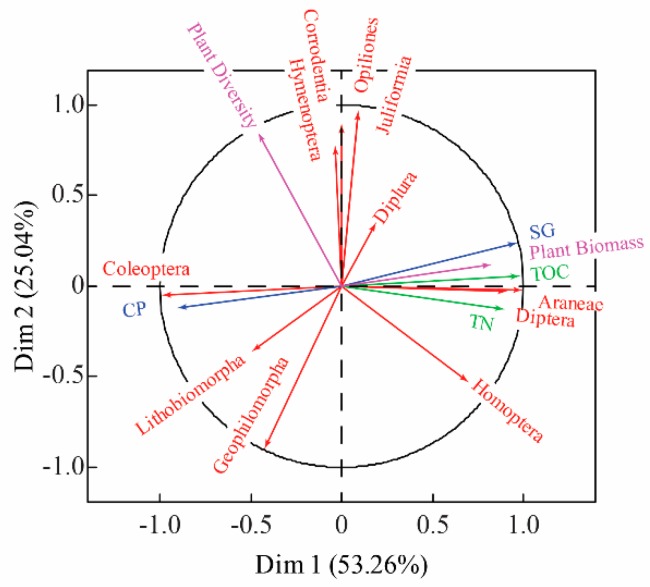
Two-dimensional ordination plot of the multiple factor analysis (MFA) performed for the entire data set, including environment factors and soil macro-arthropods data. ST: soil temperature; SM: soil moisture; TOC: soil organic matter; TN: Total N; CP: clay particle; SG: sand grains. The percentages shown next to the axis numbers are the total variations in the data explained by that axis.

A two-dimensional ordination plot of multiple factor analysis (MFA) was constructed to determine the correlations between the abundance of soil macro-arthropods and environmental factor changes caused by vegetation heterogeneity (Figure 4). There were diverse correlation patterns to environmental changes in the soil caused by vegetation heterogeneity. For instance, Coleoptera correlated positively to soil clay particle levels, and responded negatively to organic matter, total N, sand grains and plant biomass. In contrast, Araneae and Diptera were negatively correlated with the soil clay particle levels, while being positively correlated to organic matter, total N, sand grains and plant biomass. In addition, plant diversity changes caused negative responses by Homoptera, and its responses to organic matter, total N, soil clay particle, sand grains and plant biomass were slightly weaker.

**Figure 5:**
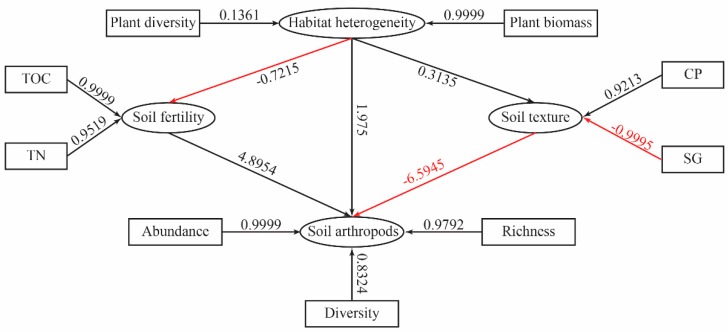
Structural equation model relating vegetation heterogeneity, soil fertility, soil texture and soil macro-arthropod communities (χ^2^ = 9.145, *p* = 0.865, GFI = 0.899, CFI = 0.907, RMSEA = 0.000). The rectangles represent the observed variables (plant diversity, biomass, soil organic matter, total N, soil clay particle, sand grains, abundance, richness and diversity); and the ovals represent latent variables (vegetation heterogeneity, soil fertility, soil texture and soil macro-arthropod communities). The red arrows indicate negative correlation between each variable; and the black arrows indicate positive correlation between each variable. The numbers indicate correlation coefficients.

The structural equation model (SEM) was designed to evaluate the correlation among the vegetation heterogeneity (plant diversity and biomass), soil fertility (soil organic matter and total N), soil texture (soil clay particle and sand grains), and soil macro-arthropods communities (abundance, richness and diversity) (Figure 5). The final model provided an excellent fit (χ^2^ = 9.145, *p* = 0.865, GFI = 0.899, CFI = 0.907, RMSEA = 0.000). The SEM model displayed a significant positive correlation between the vegetation heterogeneity and soil macro-arthropod communities (regression weight = 1.975, *p* < 0.05). This result suggests that the higher the levels of vegetation heterogeneity (plant diversity and biomass) were, the greater contribution it made to the abundance, richness and diversity of the soil macro-arthropods. At the same time, the soil macro-arthropod communities were positively affected by soil fertility (regression weight = 4.8954, *p* < 0.05). In contrast, the soil texture negatively affected soil macro-arthropod communities (regression weight = −6.5945, *p* < 0.05). In addition, we also found that soil texture was positively correlated to vegetation heterogeneity (regression weight = 0.3135, *p* < 0.05), but that soil fertility was negatively correlated with vegetation heterogeneity (regression weight = −0.7215, *p* < 0.05). These correlations suggest that vegetation heterogeneity will increase the levels of the soil clay particles, while it will decrease the soil fertility in the alpine tundra.

## 3. Discussion

### 3.1. Distribution Patterns of Soil Macro-Arthropods

Various soil macro-arthropods taxonomic compositions and distribution patterns were observed in the four plant communities to be caused by vegetation heterogeneity, and this confirmed our hypothesis that the distribution patterns of the soil macro-arthropods differ in each plant community are caused by vegetation heterogeneity (H1). Twenty-three taxa of the soil macro-arthropods (316 individuals/m^2^) were collected in the *D. angustifolia* habitat, the highest among the four habitats. Previous studies have revealed that the plant community is the key factor that can potentially affect soil macro-arthropods diversity [16,17]. Compared with the other habitats, the *D. angustifolia* habitat had the greatest richness of plant species and the most complex community structure of vegetation in the alpine tundra of the Changbai Mountains (Table 1), and this resulted in a large amount of litter on the soil surface. Previous studies have shown that abundant litter quality and species can provide abundant food and comfortable living conditions for soil macro-arthropods, as well as increasing their abundance and richness [18]. Consequently, the greatest numbers of soil macro-arthropods were found in the *D. angustifolia* habitat.

We observed that the minimum number (104 individuals/m^2^) was in the *V. uliginosum* habitat. Compared with the other habitats, the *V. uliginosum* habitat has the lowest plant diversity (0.91, Table 1). This resulted in singleness of the litter species, which consequently decreased the abundance of soil macro-arthropods [19]. In addition, the *V. uliginosum* habitat had the highest content of sand grains in this study. It is known that soil is made up of different-sized particles, and the sand grains tend to be relatively large in size (0.05–2 mm) [20]. The higher content of sand grains tends to result in greater porosity level, thus, its moisture was typically quite low [21]. Due to the higher content of sand grains in the *V. uliginosum* habitat, its soil condition is relatively drier. The moisture levels in soil are the main limiting factors of soil macro-arthropods distribution [22], and thus the minimum number (104 individuals/m^2^) was found in the *V. uliginosum* habitat.

### 3.2. Structure of the Soil Macro-Arthropod Communities 

The Venn diagram demonstrated that the four sampling sites had six taxa in common, which contributed to between 26.09% and 60.00% of the full set of taxa in each habitat (Figure 1B). At the same time, the clustering analysis revealed that there was a dissimilarity in the structure of the soil macro-arthropod communities among the different sampling sites (Figure 3). Ma and Yin found that high similarities exist in the soil arthropod communities in low elevation forests of the Changbai Mountains [23], thus, we hypothesized that there are similarities in the soil macro-arthropod communities’ structure among the different sampling sites (H2), and these results are contrary to our hypothesis. In the present study, all of the sampling sites were located in the alpine tundra of the Changbai Mountains, where the climate is much colder and windier [24]. The mobility of soil macro-arthropods is limited by the low soil temperature [25]. Perhaps due to the low soil temperature, the mobility of soil macro-arthropods is likely to be at a relatively low level, which may lead to different taxa of soil macro-arthropods concentrating at habitats that are hospitable to life. Consequently, the ratios of mutual taxa were at a lower level. Additionally, previous studies have revealed that the diversity of soil fauna in the alpine tundra is significantly lower than that in low elevation areas [26,27]. This may be another reason that the ratios of mutual taxa are relatively low.

### 3.3. Effects of Vegetation Heterogeneity on Soil Macro-Arthropods

This study observed that different orders of soil macro-arthropods could respond to changes of environmental factors caused by vegetation heterogeneity in various manners, and this confirmed our hypothesis, i.e., vegetation heterogeneity affects the different taxa of soil macro-arthropods at various levels (H3). A previous study indicated that significant differences can be found in the life history, nutrition methods, propagation characteristics, and adaptability mechanisms among different soil macro-arthropods taxa [28], thus the effects of vegetation heterogeneity on soil macro-arthropods differed among the taxa. In the present study, we found that Coleoptera correlated positively to soil clay particle levels. Coleoptera is positively correlated with soil hydrological properties [29]. Previous studies have revealed that soil clay particles are small and dense, and that it takes water much longer to move through clay soil than it does with other soil types, thus the moisture in clay soil is usually greater than other soils [30,31]. Hence, the high levels of soil clay particles may increase the abundance of Coleoptera. We also found that Araneae correlated positively with the sand grains. Because the soil macro-arthropods samples were collected from the soil surface or soil, the majority of Araneae were what would be considered “cave spiders”. The higher content of sand grains may create good conditions for cave spiders’ respiration, which may lead to Araneae correlating positively to the soil clay sand grain level.

The soil environment will change as habitats become more complex [32], and consequently the vegetation heterogeneity will alter the soil macro-arthropod communities, both directly and indirectly. In this study, the structural equation model (SEM) demonstrated that vegetation heterogeneity was positively correlated to the soil macro-arthropod communities, and affected them directly. In agreement with the “habitat heterogeneity hypothesis” [1], this result suggests that vegetation heterogeneity will increase the abundance, richness and diversity of soil macro-arthropods, and result in a greater level complexity in soil macro-arthropod communities in the alpine tundra of the Changbai Mountains. A previous study revealed that small habitat patches, which supply predator-free space or complementary resources may increase the overall quality of a pervasive habitat type, and thus lead to increases in the abundance of species [32,33]. At the same time, we observed that vegetation heterogeneity can indirectly affect the soil macro-arthropod communities by altering the soil fertility and soil texture. García-Tejero and Taboada have shown that vegetation heterogeneity enhanced the soil fertility and the decomposition process, and increased the contents of available nutrients [34]. A large amount of available nutrients could potentially promote plant growth, as well as supply sufficient food, and thus enhance the abundance, richness and diversity of soil macro-arthropods.

## 4. Materials and Methods

### 4.1. Site Descriptions

The experiment in this study was carried out in the alpine tundra located on the northern side of the Changbai Mountains, Jilin Province, China (42°02′ N, 128°03′ E). The elevation of the study area was 2200 m above sea level. This area is situated on the upper part of a volcano, and the volcanic and periglacial landforms are typically developed. The climate is a typical alpine climate, with a mean annual temperature of −7.3 °C. The mean number of snow-cover days account for more than 6 months per year. The mean annual precipitation is approximately 1100–1300 mm. The agrotype is alpine tundra soil, and the dominant species of the site were found to be *Vaccinium uliginosum*, *Sanguisorba sitchensis*, *Rhododendron aureum* and *Deyeuxia angustifolia*.

### 4.2. Sampling Design

In this study, in order to analyze the effects of the vegetation heterogeneity on soil macro-arthropod communities in the alpine tundra of the Changbai Mountains, four habitats were selected based on the previous investigation of the characteristics of the plant communities. The respective soil and vegetation characteristics of these habitats are shown in Table 1.

To avoid the extremely cold and windy climate, samples were collected in July (summer) of 2018, which also corresponds to the period of vegetative growth. Three separate stands of each habitat were randomly chosen on the Changbai Mountain West Slope at 500 m intervals. One 20 × 20 m plot was established in each stand and within each plot, and four replicates of 1 × 1 m subplots were randomly established at 5 m intervals. All plots faced west and had a slope gradient of less than 20°. Litter samples (25 × 25 cm) were taken from each subplot to collect epedaphic and hemiedaphic-living arthropods. In addition, soil core samples (25 × 25 × 10 cm), were taken to collect euedaphic-living arthropods. All litter and soil cores were thoroughly hand-sorted, and all soil macro-arthropods were collected into vials. A total of 96 soil macro-arthropods samples were collected as follows: 4 habitats × 3 replicated stands × 1 plot × 4 subplots × 2 layers. All of the samples were preserved in 75% alcohol. The arthropods were counted using an OLYMPUS SZX16 stereoscopic microscope and an OLYMPUS CX41 biomicroscope (Olympus Co., Tokyo, Japan), and were identified at the family/suborder level [35]. 

For chemical analysis, additional soil cores (10 × 10 × 10 cm) were sampled with a soil auger next to the sample site for soil macro-arthropods in each subplot. A total of 48 samples were collected as follows: 4 habitats × 3 replicated stands × 1 plot × 4 subplots. Leaves, roots, and gravel were removed from the soil samples, and each sample was air-dried and stored at room temperature. The soil properties were determined by conventional methods [36]. The organic matter was digested by K_2_Cr_2_O_7_-H_2_SO_4_, and examined using FeSO_4_ titration. At this point, the total nitrogen (N) levels were digested by H_2_SO_4_ and K_2_SO_4_-CuSO_4_∙5H_2_O-Se. The soil clay particle and sand grain contents were measured using the pipette method after soil organic matter oxidation with H_2_O_2_ and dispersion with sonication [37]. The properties of the soil in the different habitats are detailed in Table 1.

### 4.3. Statistical Analysis

In order to analyze the differences in the composition of soil macro-arthropod communities within the four habitats, the endemic taxa of each habitat were manually determined. A Venn diagram was created using a “draw-quintuple-Venn” function, available in a VennDiagram R package [38]. Generalized linear models (GLMs) were used to determine the effects of the vegetation heterogeneity on the soil macro-arthropods abundance (individuals per m^−2^) using SPSS 22 (SPSS Inc., Chicago, IL, USA).

The diversity of the soil macro-arthropod communities was quantitatively analyzed by the Shannon-Wiener index *(H′)* as follows: $H^{\prime }=-\sum_{i=1}^{s}P_{i}lnP_{i}$, where *S* is the number of species, and *Pi* the ratio of individuals in relation to the total collected individuals in species i for each habitat. This index was calculated from the pooled data of two layers from each sampling site. Rarefaction curves were computed by EstimateS 9.1.0 to compare the respective soil macro-arthropods richness and diversity of the four habitats during the sampling period [39].

An unweighted pair group method with a mathematical mean algorithm (UPGMA) was used to construct a cluster analysis of the similarities in the different soil macro-arthropod communities of each habitat. The UPGMA cluster used the “hclust” function available in the stats R package [40]. A heat map of the hierarchical clustering was created to evaluate the distribution patterns of different soil macro-arthropods taxa using a pheatmap R package [41], along with the “vegemite” function available in the vegan R package [42].

Multiple factor analysis (MFA) seeks common structures among data matrices. Such a method was applied to the data sets of plant diversity, plant biomass, soil organic matter, total N, soil clay particle, sand grains, abundance, richness and diversity of soil macro-arthropods, and to evaluate the correlations between the abundance of soil macro-arthropods and changes in environmental factors caused by vegetation heterogeneity [43]. Prior to utilizing the MFA, a Hellinger-transformation was applied to the data sets of the soil macro-arthropods [44,45]. The MFA was performed using the “MFA” function available in the FactoMineR R package [46]. 

Structural equation models (SEM) are generally used to test for direct and indirect interaction effects between independent and measured variables in a single model [47]. In the present study, SEM was used to evaluate the correlation among the vegetation heterogeneity (plant diversity and biomass), soil fertility (soil organic matter and total N), soil texture (soil clay particle and sand grains), and soil macro-arthropod communities (abundance, richness and diversity). There were nine observed variables in the model: (1) plant diversity, (2) plant biomass, (3) soil organic matter, (4) total N, (5) abundance, (6) soil clay particle, (7) sand grains, (8) richness, and (9) soil macro-arthropods diversity. The vegetation heterogeneity, soil fertility, soil texture and soil macro-arthropod communities were the latent variables in the model. In order to assess the fit of the model, χ2 tests, the goodness-of-fit index, comparative fit index and root square mean errors of approximation (RMSEA) were used [48,49]. An analysis of the structural equation model was carried out using the SEM R package [50].

## 5. Conclusions

In summary, vegetation heterogeneity affected the soil macro-arthropod communities in the alpine tundra of the Changbai Mountains. The taxonomic composition of soil macro-arthropods varied among the different habitats, and dissimilarities existed in the soil macro-arthropod communities. The abundance, richness and diversity in the *D. angustifolia* habitat were all at the maximum during the sampling period. The vegetation heterogeneity affected the different taxa of the soil macro-arthropods at various levels. Additionally, the vegetation heterogeneity had direct effects on the soil macro-arthropod communities, and also indirectly impacted the abundance, richness and diversity by altering the soil fertility and soil texture. The findings of this study have implications for the relationship between vegetation heterogeneity and soil faunal communities, and could provide assistance in developing biodiversity guidelines for alpine tundra.

## Figures and Tables

**Table 1:** Geographical information, soil and vegetation characteristics of the habitats.

Habitats	Location	Elevation (m)	Soil Organic Matter (g/kg)	Total N (g/kg)	Clay Particle (%)	Sand Grains (%)	Plant Diversity	Plant Biomass (g/m^2^)
*Vaccinium uliginosum* habitat	42°24′7.18″ N 128°5′48.99″ E	2250	27.23	9.74	6.09	39.94	0.91	1632
*Sanguisorba sitchensis* habitat	42°22′23.3″ N 128°5′53.0″ E	2260	11.87	5.99	9.75	12.22	1.36	686.4
*Rhododendron aureum* habitat	42°23′56.9″ N 128°8′20.4″ E	2253	20.33	8.78	9.68	23.41	1.14	496
*Deyeuxia angustifolia* habitat	42°24′51.8″ N 128°8′33.9″ E	2245	21.11	7.72	7.46	33.12	1.71	1268.8

## Supplementary Materials

The following are available online at https://www.mdpi.com/2223-7747/8/10/418/s1, Table S1: Abundance (ind. m^−2^) of soil arthropods in the various habitats.
