# Supplementary material for: Vegetation Heterogeneity Effects on Soil Macro-Arthropods in an Alpine Tundra of the Changbai Mountains, China
Source: Plants (Basel). 2019 Oct 16;8(10):418. doi: 10.3390/plants8100418 (PMC6843291; doi:10.3390/plants8100418)
Supplement: Supplementary file 1 [file plants-08-00418-s001.pdf]

Supplementary Material Table S1 Abundance (ind. m^{-2}) of soil arthropods in the various habitats

Order	Taxa	Habitats				Total	%
		Vaccinium uliginosum	*Sanguisorba sitchensis*	*Rhododendron aureum*	*Deyeuxia angustifolia*		
Lithobiomorpha	Lithobiidae	48	96	48	128	320	11.24
Phalangiidae	Geophilidae	48	96	96	32	272	9.55
Juliformia	Juliformia	0	0	16	64	80	2.81
Araneae	Lycosidae	48	0	16	0	64	2.25
	Agelenidae	0	16	0	16	32	1.12
	Gnaphosidae	0	0	16	0	16	0.56
	Liocranidae	0	0	0	16	16	0.56
	Anyphaenidae	32	0	32	96	160	5.62
Opiliones	Phalangiidae	0	0	0	32	32	1.12
Coleoptera	Elateridae	16	32	112	128	288	10.11
	Carabidae	48	160	48	176	432	15.17
	Scarabaeidae	0	16	16	16	48	1.69
	Cantharidae	0	0	0	16	16	0.56
	Staphyinidae	80	16	80	64	240	8.43
	Curculionidae	0	16	32	0	48	1.69
	Chrysomelidae	0	0	0	48	48	1.69
	Throscidae	0	0	0	16	16	0.56
	Cleridae	0	0	0	32	32	1.12
	Cicindelidae	0	0	0	16	16	0.56
Diptera	Brachycera	48	16	16	96	176	6.18
	Nematocera	0	0	16	0	16	0.56
	Cyclorrhapha	16	0	48	16	80	2.81
Homoptera	Aphidoidea	16	0	16	0	32	1.12
Diplura	Gampodeidae	0	0	0	32	32	1.12
	Japygidae	16	16	0	48	80	2.81

Hymenoptera	Ichenumonoidea	0	0	0	16	16	0.56
	Formicidae	0	0	80	144	224	7.87
Psocoptera	Hemipsocidae	0	0	0	16	16	0.56
	Total	416	480	688	1264	2848	
